# Methodology and enrollment findings of the Beijing adult dry eye cohort study

**DOI:** 10.3389/fmed.2026.1854836

**Published:** 2026-08-06

**Authors:** Kai Cao, Ruijia Shi, Lei Tian, Ying Jie

**Affiliations:** Beijing Institute of Ophthalmology, Beijing Tongren Hospital, Capital Medical University, Beijing, China

**Keywords:** age–sex standardization, Beijing adult dry eye cohort, China, dry eye disease, enrollment-stage findings, epidemiology, methodology, ocular surface

## Abstract

**Background/objectives:**

To describe the design, methodology, and enrollment-stage baseline findings of the Beijing Adult Dry Eye Cohort (ADEC), a population-based cohort established to investigate the epidemiology and longitudinal natural history of dry eye disease (DED) in urban Beijing.

**Methods:**

ADEC is a population-based cohort conducted in Dongcheng District, Beijing. Between 15 July and 31 August 2023, 1,249 adults were randomly selected from 15 communities using stratified cluster sampling; 1,143 completed baseline assessment (response rate 91.5%). Examinations included the Ocular Surface Disease Index (OSDI), non-invasive tear break-up time (NIBUT), tear meniscus height (TMH), meibography, lid margin evaluation, ocular redness analysis, and corneal fluorescein staining, together with demographic, systemic, lifestyle, environmental, and psychological assessments. Prevalence estimates were calculated at the participant level, and generalized estimating equations were used for inferential analyses involving eye-level ocular surface parameters.

**Results:**

The baseline analytic sample included 1,091 participants (mean age 54.8 ± 18.0 years; 62.6% female), with eye-level ocular surface measurements available for up to 2,180 eyes depending on the parameter assessed. The crude prevalence of DED, defined as an OSDI score ≥13 together with average NIBUT <10 s in either eye, was 33.7%. After age–sex standardization to the 2020 Chinese Census, the prevalence was 27.0% (95% CI: 23.8–30.3%). The crude prevalence of SDE, aqueous-deficient (AD) phenotype, and MGD phenotype was 34.7, 33.6, and 31.0%, respectively. Mean average NIBUT was 12.0 ± 4.2 s and mean TMH was 0.19 ± 0.07 mm. Participant-level average NIBUT was negatively correlated with anxiety (Spearman *r* = −0.535, *p* < 0.001) and depression (Spearman *r* = −0.425, *p* < 0.001). Participants with daily electronic device use >2 h comprised 71.0% of the cohort, and 5.1% had autoimmune or rheumatic disease.

**Conclusion:**

These enrollment-stage ADEC findings characterize the burden and ocular surface profile of dry eye disease among community-dwelling adults in urban Beijing and provide a foundation for subsequent longitudinal analyses within the cohort.

## Introduction

Adult dry eye disease (DED) is a chronic ocular surface inflammatory disorder arising from dysregulation within a multimodal pathological network, demonstrating spatiotemporal heterogeneity in its pathogenesis characterized by multi-system interactions (1). From a systems medicine perspective, the pathogenic elements can be deconstructed into four dynamically coupled dimensions: (1) Environmental Exposome: Aerodynamic studies demonstrate that wind speed and humidity significantly impact tear film stability, with decreased relative humidity leading to shortened tear film break-up time (TBUT) (2). Particulate matter (PM2.5) exposure triggers corneal inflammation through activation of the NLRP3 inflammasome or reactive oxygen species/p38 MAPK pathway (3, 4). (2) Behavioral Exposome: Visual display terminal (VDT) usage duration exhibits a dose–response relationship with TBUT, with potential mechanisms involving parasympathetic inhibition (5–8). (3) Metabolic-Immune Axis: Advanced glycation end-products (AGEs) deposition in diabetic patients promotes goblet cell apoptosis via the RAGE/NF-κB pathway, while Th17/Treg imbalance in rheumatoid arthritis induces IL-17-mediated lacrimal duct epithelial damage (9–11). (4) Ocular Surface Microbiome: Emerging evidence suggests that gut and ocular surface microbiota modifications may influence dry eye symptoms through modulation of inflammatory cytokines (12, 13).

Cohort studies, recognized as an important design for characterizing spatiotemporal dynamics of disease progression, leverage their longitudinal design to comprehensively deconstruct phenotypic heterogeneity, gene–environment interactions, and temporal associations for future longitudinal analyses. However, current ophthalmic epidemiological cohorts face three critical methodological constraints: First, study design exhibits target deviation—landmark large-scale cohorts (e.g., the Beaver Dam Eye Study (14), the Handan Eye Study (15), Singapore Chinese Eye Study (16), the Kumejima Study (17), and Blue Mountains Eye Study (18)) predominantly focus on glaucoma or retinopathy as primary endpoints, relegating dry eye assessments to secondary outcomes with incomplete measurement. Second, exposomic frameworks remain deficient, failing to integrate quantified models of VDT usage or implement spatiotemporally resolved monitoring of aerodynamical parameters. Third, significant sampling bias persists among critical pathophysiological subpopulations: key cohorts including perimenopausal women, occupational digital natives with chronic screen-associated desiccating stress, and autoimmune-predisposed carriers demonstrate marked underrepresentation in extant datasets.

These systemic deficiencies have engendered global evidence gaps in DED epidemiology. A 2021 systematic review in JAMA Ophthalmology revealed that only two U.S. studies reported DED incidence, one predating smartphone ubiquity (19). Data from other countries and regions, such as China (20), Japan (21), and the United Kingdom (22), are even more scarce. Establishing systematic and standardized dry eye disease cohorts is of immense scientific and clinical significance. This is the impetus for launching the Beijing Adult Dry Eye Cohort (ADEC) study.

This study establishes a dedicated population-based dry eye cohort in urban Beijing and implements a comprehensive assessment framework encompassing environmental, behavioral, systemic, and psychological dimensions, surpassing the limitations of previous cohorts focused primarily on other ocular diseases. With a prospectively designed biospecimen repository, we have systematically collected and stored tear fluid samples, laying a crucial foundation for future tear lipidomics, metagenomics, and proteomics analyses, thereby providing unique value for exploring gene–environment-behavior relationships and future mechanistic analyses. The cohort specifically includes key subpopulations relevant to DED research, such as perimenopausal women and individuals with high digital device exposure, aligning with contemporary disease patterns. This article systematically describes the cohort’s methodological framework and reports enrollment-stage baseline findings.

## Materials and methods

### Study design and setting

The Beijing Adult Dry Eye Cohort (ADEC) is a population-based longitudinal cohort study conducted in Dongcheng District, Beijing, China. The present manuscript reports enrollment-stage baseline cross-sectional findings from cohort enrollment. Beijing city, located between 115.7° and 117.4° east longitude and 39.4°–41.6° north latitude, is the capital and municipality of China. In 2025, the per capita gross domestic product (GDP) of Beijing was approximately 239,000 yuan (about 33,000 USD), representing the economic development level of a major metropolitan area in China (23).

Baseline recruitment was conducted between July 15 and August 31, 2023. Follow-up examinations are part of the longitudinal ADEC design; however, the present analysis is restricted to baseline data.

### Sampling strategy

The study employed a stratified cluster random sampling method to select participants. First, three streets (Jian Guo Men Street, Jiao Dao Kou Street, and Yong Ding Men Wai Street) representing the northern, central, and southern parts of Dongcheng District were selected. Within these streets, 15 communities were stratified based on geographic location, population density, and socioeconomic status. Adult participants were then randomly sampled from each selected community, with the number of participants proportional to the community’s adult population. A total of 1,249 adults were initially enrolled, of whom 1,143 completed the baseline assessment, yielding a response rate of 91.5%.

### Ethical approval

The study was approved by the Ethics Committee of Beijing Tongren Hospital, Capital Medical University (approval No. TREC2024-KY084). This study adhered to the principles of the Declaration of Helsinki. Written informed consent was obtained from all participants after explanation of the nature and possible consequences of the study. The trial was registered with ClinicalTrials.gov (NCT06621446) on 15 July 2023.

### Inclusion and exclusion criteria

Inclusion criteria were: (1) Aged 18 years or older; (2) Resident of the selected community for at least 6 months; (3) Able to undergo and complete a comprehensive dry eye examination; (4) Signed informed consent. Exclusion criteria were: (1) Diagnosed with severe mental illness or cognitive impairments affecting participation; (2) Severe mobility impairments preventing participation in examinations; (3) History of ocular surgery or any condition that could interfere with dry eye evaluation; (4) Unable or unwilling to participate in follow-up assessments.

### Outcomes and measures

A dry eye and corneal topography tester (MediWorks, Shanghai, China, DEA520) was used to perform a comprehensive examination of dry eye. Clinical assessments included: (1) Ocular Surface Disease Index (OSDI) questionnaire in Chinese version; (2) non-invasive tear break-up time (NIBUT); (3) tear meniscus height (TMH); (4) infrared meibography for meibomian gland evaluation; (5) lid margin evaluation; (6) ocular redness analysis; and (7) corneal fluorescein sodium staining (CFS). NIBUT and TMH were automatically measured by the device. Both eyes of each participant were examined, and eye-level analyses of objective ocular surface indicators accounted for inter-eye correlation within participants. Except for the above measurements, tear samples were also collected by trained investigators in a controlled environment. Non-stimulated tear fluid was collected using sterile, additive-free standard Schirmer strips. The bent tip (0 mm mark) of the strip was gently placed in the lateral one-third of the lower conjunctival sac, and participants were instructed to close their eyes gently. After 5 min, the moistened strip was carefully removed and immediately placed, using sterile forceps, into a pre-labeled 1.5 mL sterile microcentrifuge tube containing 100 μL of phosphate-buffered saline with protease inhibitors. Within 30 min of collection, samples were centrifuged at 4 °C and 12,000×*g* for 10 min to elute tear components and remove debris. The supernatant was then aliquoted using low-retention pipette tips into multiple pre-labeled 0.2 mL microtubes to avoid repeated freeze–thaw cycles. All aliquots were snap-frozen in liquid nitrogen within 2 h of collection and subsequently transferred to a −80 °C ultra-low temperature freezer for long-term storage. All samples are linked to detailed metadata and managed under strict temperature monitoring and access logging to ensure sample quality and traceability.

### Dry eye definition

Dry eye was assessed using the following participant-level definitions:

1. Dry eye disease (DED): OSDI score ≥13 and average NIBUT <10 s in either eye.
2. Symptomatic Dry Eye (SDE): OSDI score ≥13.
3. Aqueous-deficient phenotype (AD phenotype): OSDI score ≥13 and TMH < 0.2 mm in either eye.
4. MGD phenotype: OSDI score ≥13 together with average NIBUT <13 s and a summed upper and lower meibomian gland score >2 in either eye.

### Data collection

Detailed information on demographic characteristics, systemic diseases, lifestyle factors, environmental factors, and psychological status was collected through structured questionnaires before ocular examinations. Systemic diseases included diabetes, hypertension, hyperlipidemia, coronary artery disease, stroke, and rheumatic disease. Lifestyle factors included smoking, alcohol consumption, electronic device usage, dietary habits (vitamin A, vegetable, and fruit intake), and sleep quality. Environmental exposures included long-term indoor/outdoor work, dust exposure, and heated environment exposure. Psychological status was assessed using the 14-item Hamilton Anxiety Rating Scale (HAMA) and the 17-item Hamilton Depression Rating Scale (HAMD).

### Quality control

Before the start of the study, unified training was conducted for all inspectors, including questionnaire administration and eye examination techniques. The consistency within and between surveyors was evaluated. Intraclass correlation coefficient (ICC) values or weighted Kappa values were above 0.8, indicating satisfactory agreement.

### Statistical analysis

Statistical analysis was conducted using R software (version 4.3.0). Continuous variables were described as mean ± standard deviation (SD) or median and range as appropriate. Categorical variables were described as frequencies and percentages. The primary prevalence outcome was DED; SDE, AD phenotype, and MGD phenotype were summarized as secondary dry eye-related phenotypes. Prevalence estimates for DED, SDE, AD phenotype, and MGD phenotype were calculated at the participant level using the available denominator for each outcome, with exact binomial 95% confidence intervals (CIs). Different denominators reflect variable-specific missing data required for each outcome definition. For inferential analyses involving eye-level ocular surface parameters, generalized estimating equations (GEE) with a Gaussian distribution, identity link, exchangeable working correlation structure, and robust standard errors were used to account for inter-eye correlation within participants; participant identifier was used as the clustering variable. Exploratory correlations between continuous factors and dry eye parameters were assessed at the participant level using Spearman correlation coefficients. For NIBUT and TMH in participant-level correlation analyses, bilateral eye measurements were summarized as participant-level averages. Cross-sectional associations with selected binary factors were assessed using participant-level linear models. All analyses were interpreted as baseline cross-sectional associations.

### Age–sex standardization

Age–sex standardized prevalence rates were calculated using the direct standardization method with the 2020 Chinese Census population as the standard population. Standardization was performed using sex-specific age strata. Because the Census table reports a 15–19-year age group, 40% of this stratum was used to approximate the 18–19-year standard population to match the study eligibility criterion of age ≥18 years. The ≥80-year standard stratum combined all Census age groups aged 80 years and older. Standardized rates were calculated as the sum of weighted stratum-specific prevalence estimates.

## Results

### Response rate and baseline analytic sample

Of the 1,249 adults randomly selected from 15 communities, 1,143 completed the baseline assessment, yielding a response rate of 91.5%. After excluding participants with incomplete baseline data required for the main dry eye analyses, the baseline analytic sample included 1,091 participants. Eye-level ocular surface measurements were available for up to 2,180 eyes, depending on the parameter assessed.

### Demographic characteristics

The participant-level demographic and clinical characteristics of the 1,091 participants are summarized in Table 1. The mean age was 54.8 ± 18.0 years (median: 61.0 years, range: 18–90 years), with participants stratified into three age groups: 18–39 years (266 cases, 24.4%), 40–59 years (259 cases, 23.7%), and ≥60 years (566 cases, 51.9%). Female participants accounted for 62.6% (683 cases) of the total, with a higher female proportion in the 40–59 years (69.9%) and ≥60 years (67.0%) groups compared to the 18–39 years group (46.2%). The mean OSDI score was 10.7 ± 9.35 (median: 8.00, range: 0–69.0), with OSDI scores increasing across age groups (18–39 years: 6.47 ± 6.17; 40–59 years: 10.6 ± 9.43; ≥60 years: 12.7 ± 9.89).

**Table 1:** Participant-level demographic and clinical characteristics.

Characteristic	18–39 years (*n* = 266)	40–59 years (*n* = 259)	≥60 years (*n* = 566)	Total (*n* = 1,091)
Age (years)				
Mean (SD)	28.2 (6.42)	50.0 (5.60)	69.4 (5.91)	54.8 (18.0)
Median [Min, Max]	27.0 [18.0, 39.0]	50.0 [40.0, 59.0]	69.0 [60.0, 90.0]	61.0 [18.0, 90.0]
Sex (*n*, %)				
Male	143 (53.8%)	78 (30.1%)	187 (33.0%)	408 (37.4%)
Female	123 (46.2%)	181 (69.9%)	379 (67.0%)	683 (62.6%)
OSDI score				
Mean (SD)	6.47 (6.17)	10.6 (9.43)	12.7 (9.89)	10.7 (9.35)
Median [Min, Max]	4.00 [0, 33.0]	8.00 [0, 69.0]	10.0 [0, 56.0]	8.00 [0, 69.0]
First NIBUT (s)				
Mean (SD)	12.5 (4.60)	10.7 (4.63)	9.39 (4.29)	10.5 (4.62)
Median [Min, Max]	11.3 [3.47, 26.9]	10.6 [1.61, 21.3]	10.0 [0.380, 23.0]	10.5 [0.380, 26.9]
Average NIBUT (s)^*^				
Mean (SD)	13.9 (4.05)	12.3 (4.34)	11.0 (3.96)	12.0 (4.24)
Median [Min, Max]	13.2 [5.17, 26.9]	11.9 [2.64, 26.7]	11.2 [2.72, 23.0]	11.9 [2.64, 26.9]
Tear meniscus height (mm)^*^				
Mean (SD)	0.184 (0.0549)	0.196 (0.0764)	0.178 (0.0707)	0.184 (0.0690)
Median [Min, Max]	0.180 [0.100, 0.580]	0.180 [0.0700, 0.540]	0.167 [0.0500, 0.690]	0.170 [0.0500, 0.690]

^*^For NIBUT and TMH in this table, bilateral eye measurements were summarized at the participant level.

### Dry eye prevalence

Based on the study’s participant-level dry eye definitions, the crude prevalence of DED and dry eye-related phenotypes varied by sex and age (Table 2). The overall crude prevalence of DED was 33.73%. The crude prevalence was 34.74% for SDE, 33.58% for AD phenotype, and 31.01% for MGD phenotype. Females had consistently higher crude prevalence than males across DED and all dry eye-related phenotypes (DED: 41.00% vs. 21.57%; SDE: 42.02% vs. 22.55%; AD phenotype: 40.76% vs. 21.57%; MGD phenotype: 37.52% vs. 20.25%). For both sexes, prevalence generally increased with age.

**Table 2:** Participant-level crude prevalence of DED and dry eye-related phenotypes by sex and age group.

Sex	Age group	Total *n*	DED, %	SDE, %	AD phenotype, %	MGD phenotype, %
Female	18–39 years	123	24.39	26.02	25.20	17.80
Female	40–59 years	181	37.02	37.02	36.67	31.98
Female	≥60 years	379	48.28	49.60	47.76	46.56
Female	Total	683	41.00	42.02	40.76	37.52
Male	18–39 years	143	6.99	6.99	6.99	5.04
Male	40–59 years	78	24.36	25.64	24.36	19.74
Male	≥60 years	187	31.55	33.16	31.55	32.22
Male	Total	408	21.57	22.55	21.57	20.25
Total	Total	1,091	33.73	34.74	33.58	31.01

Prevalence values are crude percentages calculated at the participant level. DED was defined as an OSDI score ≥13 together with average NIBUT <10 s in either eye. SDE was defined as an OSDI score ≥13. AD phenotype was defined as an OSDI score ≥13 together with TMH <0.2 mm in either eye. MGD phenotype was defined as an OSDI score ≥13 together with average NIBUT <13 s and a summed upper and lower meibomian gland score >2 in either eye. Different denominators reflect variable-specific missing data required for each outcome definition. Abbreviations: AD, aqueous-deficient; DED, dry eye disease; MGD, meibomian gland dysfunction; SDE, symptomatic dry eye.

After age–sex standardization using the 2020 Chinese Census population, the standardized prevalence and 95% confidence intervals (CI) were calculated for DED and dry eye-related phenotypes (Table 3). The standardized prevalence was 27.04% (95% CI: 23.81–30.26%) for DED, 27.84% (95% CI: 24.58–31.09%) for SDE, 26.95% (95% CI: 23.73–30.18%) for AD phenotype, and 22.44% (95% CI: 19.49–25.83%) for MGD phenotype.

**Table 3:** Crude and age–sex standardized prevalence of DED and dry eye-related phenotypes.

Outcome	Available *N*	Cases	Crude prevalence	Crude 95% CI	Age–sex standardized prevalence	Standardized 95% CI	Missing *N*
DED	1,091	368	33.73%	30.93–36.62%	27.04%	23.81–30.26%	0
SDE	1,091	379	34.74%	31.91–37.65%	27.84%	24.58–31.09%	0
AD phenotype	1,090	366	33.58%	30.78–36.47%	26.95%	23.73–30.18%	1
MGD phenotype	1,048	325	31.01%	28.22–33.91%	22.44%	19.49–25.83%	43

Prevalence estimates were calculated at the participant level. DED was defined as an OSDI score ≥13 together with average NIBUT <10 s in either eye. SDE was defined as an OSDI score ≥13. AD phenotype was defined as an OSDI score ≥13 together with TMH <0.2 mm in either eye. MGD phenotype was defined as an OSDI score ≥13 together with average NIBUT <13 s and a summed upper and lower meibomian gland score >2 in either eye. Different denominators reflect variable-specific missing data required for each outcome definition. Crude 95% confidence intervals were calculated using the exact binomial method. Age–sex standardized prevalence was calculated by direct standardization to the 2020 Chinese Census population using sex-specific age strata.

## Objective ocular surface indicators

### Non-invasive tear break-up time (NIBUT)

NIBUT showed significant differences by age group and sex in GEE analyses accounting for inter-eye correlation (Table 4). The overall mean average NIBUT was 12.0 ± 4.25 s. Mean average NIBUT decreased across age groups (18–39 years: 13.80 ± 3.99 s; 40–59 years: 12.16 ± 4.22 s; ≥60 years: 11.05 ± 4.09 s; GEE p for age group <0.001) and was higher in males than females (13.00 ± 4.22 s vs. 11.38 ± 4.15 s; GEE p for sex <0.001).

**Table 4:** Eye-level NIBUT distribution by age group and sex.

Grouping factor	Group	*N* eyes	*N* participants	First NIBUT, mean ± SD (s)	Average NIBUT, mean ± SD (s)	GEE *p* value
Age group	18–39 years	531	266	12.47 ± 4.51	13.80 ± 3.99	<0.001
Age group	40–59 years	518	259	10.59 ± 4.57	12.16 ± 4.22	
Age group	≥60 years	1,131	566	9.50 ± 4.38	11.05 ± 4.09	
Sex	Female	1,365	683	9.83 ± 4.44	11.38 ± 4.15	<0.001
Sex	Male	815	408	11.59 ± 4.70	13.00 ± 4.22	
Overall	Total	2,180	1,091	10.49 ± 4.61	11.98 ± 4.25	

Values are presented at the eye level. Inferential analyses accounted for inter-eye correlation using generalized estimating equations (GEE). The GEE p values indicate overall age-group or sex differences in NIBUT parameters.

### Tear meniscus height (TMH)

TMH and tear meniscus area also differed by age group and sex in eye-level GEE analyses (Table 5). The overall mean TMH was 0.184 ± 0.069 mm. Males had a higher mean TMH than females (0.193 ± 0.076 mm vs. 0.180 ± 0.071 mm; GEE p for sex = 0.002). Across age groups, mean TMH was highest in the 40–59 years group (0.198 ± 0.084 mm; GEE p for age group = 0.001). Tear meniscus area showed a similar pattern, with significant variation by age group (GEE *p* < 0.001) and sex (GEE *p* = 0.023).

**Table 5:** Eye-level tear meniscus parameters by age group and sex.

Grouping factor	Group	*N* eyes	*N* participants	TMH, mean ± SD (mm)	Tear meniscus area, mean ± SD	GEE *p* value
Age group	18–39 years	529	266	0.184 ± 0.056	1.77 ± 0.65	TMH: 0.001; area: <0.001
Age group	40–59 years	516	258	0.198 ± 0.084	2.07 ± 0.90	
Age group	≥60 years	1,129	566	0.179 ± 0.074	1.80 ± 0.80	
Sex	Female	1,360	682	0.180 ± 0.071	1.83 ± 0.81	TMH: 0.002; area: 0.023
Sex	Male	814	408	0.193 ± 0.076	1.90 ± 0.78	

### Additional ocular surface signs

Additional baseline ocular surface signs, including CFS, lid margin status, ocular redness, and meibomian gland scores, are summarized in Table 6.

**Table 6:** Baseline additional ocular surface signs.

Variable	Available data	Summary	Missing
CFS score	1,844 eyes/933 participants	0.44 ± 1.13; median 0.00 (0.00, 1.00)	336 eyes
CFS positive	1,844 eyes/933 participants	469 (25.4%)	336 eyes
Upper lid margin score	1,167 eyes/612 participants	1.00 ± 0.00	1,013 eyes
Lower lid margin score	1,165 eyes/598 participants	1.01 ± 0.10	1,015 eyes
Any lid margin abnormality	1,186 eyes/616 participants	11 (0.9%)	994 eyes
Overall redness mean	2,168 eyes/1,087 participants	0.14 ± 0.03	12 eyes
Upper meibomian gland score	2,078 eyes/1,052 participants	1.60 ± 0.60	102 eyes
Lower meibomian gland score	2,115 eyes/1,066 participants	1.81 ± 0.76	65 eyes

Values are presented at the eye level. Lid margin abnormality was defined as a lid margin score greater than 1, with a score of 1 indicating normal findings. Abbreviations: CFS, corneal fluorescein staining.

### Key subpopulations for DED research

The ADEC cohort includes participants with lifestyle and systemic characteristics relevant to future DED research (Table 7). Daily electronic device use >2 h was reported by 71.04% of participants (775 cases), and 5.13% (56 cases) had autoimmune or rheumatic diseases. These subgroups provide a basis for future longitudinal analyses of lifestyle-related and autoimmune-associated dry eye phenotypes.

**Table 7:** Distribution of key subpopulations for future DED research (*N* = 1,091).

Indicator	Number	Percentage
Digital natives (daily electronic device use >2 h)	775	71.04%
Autoimmune/rheumatic disease patients	56	5.13%

### Chronic diseases, lifestyle and environmental exposures

The prevalence of common chronic systemic diseases, lifestyle factors, and environmental exposures in the cohort is summarized in Table 8. The most prevalent chronic diseases were hyperlipidemia (33.18%), hypertension (31.07%), myopia (41.61%), and diabetes (19.07%); cardiovascular diseases included coronary artery disease (11.46%) and stroke (3.76%). For lifestyle factors, 16.96% of participants were smokers and 15.31% were regular alcohol consumers. Environmental exposures included long-term indoor work (47.66%), long-term dust exposure (6.87%), and long-term heated environment exposure (20.26%).

**Table 8:** Prevalence of chronic diseases, lifestyle, and environmental exposures.

Indicator	*n* (%)
Diabetes	208 (19.07%)
Hypertension	339 (31.07%)
Hyperlipidemia	362 (33.18%)
Coronary artery disease	125 (11.46%)
Stroke	41 (3.76%)
Myopia	454 (41.61%)
Smoking	185 (16.96%)
Regular alcohol consumption	167 (15.31%)
Long-term indoor work	520 (47.66%)
Long-term dust exposure	75 (6.87%)
Long-term heated environment exposure	221 (20.26%)

In GEE models that accounted for inter-eye correlation, average NIBUT was cross-sectionally associated with sex, age, myopia, oral mucosal dryness, electronic device use >2 h/day, and psychological status. In the HAMA model (2,131 eyes from 1,066 participants), male sex was associated with higher average NIBUT (*β* = 0.810, 95% CI: 0.372–1.248; *p* < 0.001), whereas older age (*β* = −0.049 per year, 95% CI: −0.060 to −0.038; *p* < 0.001), myopia (*β* = −0.531, 95% CI: −0.956 to −0.105; *p* = 0.015), oral mucosal dryness (*β* = −1.098, 95% CI: −1.528 to −0.667; *p* < 0.001), electronic device use >2 h/day (*β* = −0.855, 95% CI: −1.322 to −0.388; *p* < 0.001), and HAMA score (*β* = −0.285, 95% CI: −0.347 to −0.223; *p* < 0.001) were associated with lower average NIBUT. Similar associations were observed in the HAMD model.

### Psychological status and exploratory correlation analyses

The mean HAMA score was 6.66 ± 4.95 and the mean HAMD score was 7.69 ± 3.34 (Table 9). Exploratory participant-level analyses showed that age, HAMA score, and HAMD score were positively correlated with OSDI and negatively correlated with participant-level average NIBUT and TMH (Table 10). Participant-level average NIBUT was negatively correlated with HAMA (Spearman *r* = −0.535, *p* < 0.001) and HAMD (Spearman *r* = −0.425, *p* < 0.001). Electronic device use >2 h/day was associated with higher OSDI, lower participant-level average NIBUT, and lower participant-level TMH.

**Table 9:** Psychological scale scores.

Psychological Scale	Mean ± SD
Hamilton Anxiety Rating Scale (HAMA)	6.66 ± 4.95
Hamilton Depression Rating Scale (HAMD)	7.69 ± 3.34

**Table 10:** Exploratory participant-level correlation and association analyses.

Analysis	Outcome	Factor	*N*	Estimate	95% CI	*p* value
Spearman correlation	OSDI	Age	1,091	*r* = 0.295	–	<0.001
Spearman correlation	OSDI	HAMA	1,090	*r* = 0.558	–	<0.001
Spearman correlation	OSDI	HAMD	1,090	*r* = 0.454	–	<0.001
Spearman correlation	Participant-average NIBUT	Age	1,091	*r* = −0.274	–	<0.001
Spearman correlation	Participant-average NIBUT	HAMA	1,090	*r* = −0.535	–	<0.001
Spearman correlation	Participant-average NIBUT	HAMD	1,090	*r* = −0.425	–	<0.001
Spearman correlation	Participant-average TMH	Age	1,090	*r* = −0.120	–	<0.001
Spearman correlation	Participant-average TMH	HAMA	1,089	*r* = −0.272	–	<0.001
Spearman correlation	Participant-average TMH	HAMD	1,089	*r* = −0.211	–	<0.001
Binary association	OSDI	Electronic device >2 h/day	1,068	*β* = 4.284	3.053–5.514	<0.001
Binary association	OSDI	Myopia	1,072	*β* = 1.012	−0.116 to 2.140	0.079
Binary association	OSDI	Dust exposure	1,073	*β* = 2.650	0.458–4.843	0.018
Binary association	Participant-average NIBUT	Electronic device >2 h/day	1,068	*β* = −1.666	−2.183 to −1.149	<0.001
Binary association	Participant-average NIBUT	Myopia	1,072	*β* = −0.112	−0.587 to 0.363	0.644
Binary association	Participant-average NIBUT	Dust exposure	1,073	*β* = −0.491	−1.412 to 0.430	0.296
Binary association	Participant-average TMH	Electronic device >2 h/day	1,067	*β* = −0.011	−0.020 to −0.003	0.010
Binary association	Participant-average TMH	Myopia	1,071	*β* = 0.005	−0.003 to 0.013	0.188
Binary association	Participant-average TMH	Dust exposure	1,072	*β* = −0.015	−0.030 to 0.001	0.060

NIBUT and TMH were summarized as participant-level bilateral averages for correlation analyses. *β* represents the mean difference for group 1 versus group 0 in participant-level models.

## Discussion

The Beijing Adult Dry Eye Cohort (ADEC) is a population-based, disease-specific cohort dedicated to dry eye disease (DED) in urban Beijing. This study presents detailed enrollment-stage baseline epidemiological data of DED among community-dwelling adults in urban Beijing, with rigorous age–sex standardization, comprehensive ocular surface assessments, and systematic collection of systemic, lifestyle, psychological, and environmental data. The enrollment-stage baseline results show that DED was common in urban Beijing and was associated with age, sex, psychological status, myopia, and modern lifestyle factors, supporting DED epidemiological research in China and providing a foundation for future longitudinal analyses.

### DED prevalence: crude vs. standardized rates and regional comparisons

The crude prevalence of DED in the ADEC cohort was 33.73%, with SDE at 34.74%, AD phenotype at 33.58%, and MGD phenotype at 31.01%—rates that are consistent with the crude prevalence of DED in China reported in a recent database-driven study (41.8%) (24), and higher than the overall DED prevalence in Asian populations (23.9%) (24). After age–sex standardization using the 2020 Chinese Census population, the standardized prevalence of DED decreased to 27.04% (SDE: 27.84%, AD phenotype: 26.95%, MGD phenotype: 22.44%), which is within the global DED prevalence range (5–50%) reported in a systematic review (1), and aligns with the latest Asian DED prevalence estimates (23.9%) (24). The discrepancy between crude and standardized rates is consistent with the overrepresentation of adults aged ≥60 years (51.9%) in the ADEC cohort, a group with the highest DED prevalence (≥33% for SDE in both sexes). This finding highlights the critical importance of age–sex standardization for cross-study DED prevalence comparisons, as unstandardized crude rates can be biased by the age and sex structure of the study population.

Notably, the standardized prevalence of MGD phenotype in the ADEC cohort was 22.44%, lower than but closer to the standardized estimates for SDE and AD phenotype. Differences from some Western studies that report MGD as the most common DED subtype (19), may reflect variation in diagnostic definitions, ethnicity, environmental conditions, age structure, and meibomian gland assessment methods. For example, the high proportion of digital natives (71.04%) in the ADEC cohort may be associated with aqueous-deficient features through reduced blink rate and tear evaporation associated with prolonged screen use (5, 25), while MGD, which is more closely linked to sebaceous gland dysfunction and hormonal factors (26), showed a different prevalence pattern in this cohort. In addition, the phenotype-specific criteria for MGD in this study incorporated symptoms, tear-film instability, and summed meibomian gland score criteria.

### Age and sex: core demographic correlates of DED

Consistent with previous global and Asian studies (1, 24, 26), the ADEC results show that age and sex were core demographic correlates of DED, with DED prevalence increasing significantly with age and females having a much higher DED prevalence than males. The ≥60 years age group had the highest DED prevalence (female SDE:49.60%, male SDE:33.16%), which may reflect age-related degenerative changes in the ocular surface: lacrimal gland hypofunction leading to reduced tear secretion (27), progressive meibomian gland atrophy and dysfunction (28), and decreased corneal epithelial cell turnover (1). In addition, older adults have a higher prevalence of chronic systemic diseases (e.g., diabetes, hypertension) (Table 8), which may further affect ocular surface status and tear film stability (9, 10).

Females had a 2-fold higher crude SDE prevalence than males (42.02% vs. 22.55%), a finding consistent with the TFOS DEWS II sex and hormones report (26) and Asian population studies (24). The underlying mechanisms are closely related to hormonal fluctuations in females: androgens, which play a key role in maintaining meibomian gland lipid synthesis and lacrimal gland function (26), are present at lower levels in females; during perimenopause and postmenopause, estrogen and progesterone decline further, leading to reduced tear secretion, increased tear evaporation, and ocular surface inflammation (26). The ADEC cohort found that females had significantly lower NIBUT (11.38 ± 4.15 s vs. 13.00 ± 4.22 s in males) and TMH (0.180 ± 0.071 mm vs. 0.193 ± 0.076 mm in males), which are consistent with poorer tear film stability in females and provide objective ocular evidence supporting sex-related differences in DED.

### Psychological factors: a critical correlate of tear film stability

The ADEC study found significant negative correlations between anxiety/depression and average NIBUT (*r* = −0.535 and *r* = −0.425, both *p* < 0.001), and GEE and exploratory participant-level analyses showed associations between higher HAMA and HAMD scores and reduced NIBUT—findings that support the understanding of the bidirectional relationship between mental health and DED (29, 30). A recent meta-analysis (29) reported that the prevalence of anxiety and depression in DED patients is three times higher than in the general population, and the ADEC results suggest that even subthreshold anxiety/depression (mean HAMA:6.66 ± 4.95, mean HAMD:7.69 ± 3.34, below the clinical diagnosis threshold) may be associated with tear film instability.

The bidirectional interaction between psychological factors and DED may involve three mechanisms: (1) DED-induced psychological distress: chronic ocular discomfort (dryness, foreign body sensation, blurred vision) impairs daily life and quality of life, potentially contributing to anxiety and depression, especially in patients with persistent symptoms (30); (2) Psychological distress associated with DED severity: anxiety and depression may increase ocular surface sensory nerve sensitivity (22), reduce the discomfort threshold, and be associated with increased subjective DED symptoms; in addition, stress activates the sympathetic nervous system, which may be associated with reduced blink rate and tear secretion (29); (3) Somatization: more than 80% of depressed individuals have somatization symptoms (30), which may amplify subjective DED symptoms and cause a disconnect between DED symptoms and objective signs. The ADEC findings highlight the need for a multidisciplinary approach to DED management: in addition to ophthalmic treatment, psychological assessment and intervention may be considered in comprehensive assessment of DED patients, especially those with severe symptoms and normal objective indicators.

### Myopia and chronic systemic diseases: relevant clinical correlates

The ADEC study observed that myopia (41.61% prevalence) was associated with reduced NIBUT in eye-level GEE models, a finding that has received little attention in previous DED epidemiological studies. Myopia, especially high myopia, is associated with ocular structural changes: axial elongation leads to stretching of the ocular surface tissues, including the lacrimal gland and meibomian glands, which could affect ocular surface physiology; in addition, myopic patients are more likely to have prolonged near vision (e.g., reading, digital device use), which further reduces blink rate and exacerbates tear film instability (5). This finding is particularly important for China, where the myopia prevalence is the highest in the world (over 50% in adults) (31), and suggests that myopia should be considered as a potential correlate or confounder in future DED research.

Common chronic systemic diseases (diabetes:19.07%, hypertension:31.07%, hyperlipidemia:33.18%) were highly prevalent in the ADEC cohort, consistent with the age structure (51.9% ≥ 60 years). Diabetes is a well-recognized condition associated with DED (9), as advanced glycation end-products (AGEs) deposit in the ocular surface and induce goblet cell apoptosis via the RAGE/NF-κB pathway, leading to reduced mucin secretion and tear film instability (9). Hypertension and hyperlipidemia may be associated with microcirculatory changes affecting the lacrimal gland and meibomian glands (32). Although the baseline analyses did not establish causal relationships between these chronic diseases and NIBUT, their high prevalence supports the importance of collecting systemic disease data and considering systemic comorbidities in future DED cohort analyses.

### Digital natives: a dominant modern lifestyle exposure

The ADEC cohort found that 71.04% of participants were digital natives (daily electronic device use >2 h), a proportion much higher than in Western cohorts (19) and reflecting the rapid digitalization of work and daily life in urban China. Prolonged digital device use is a established exposure associated with DED (5, 25), with proposed mechanisms including: (1) Reduced blink rate and incomplete blinking: normal blink rate is 15–20 times/min, while screen use reduces it to 5–10 times/min, which may increase tear evaporation (5); (2) Ocular surface desiccation: prolonged gaze at screens causes the eyes to open wider, increasing the exposed ocular surface area and accelerating tear evaporation (25); (3) Blue light exposure: digital device blue light may induce oxidative stress in the corneal and conjunctival epithelial cells, leading to ocular surface inflammation (33).

A systematic review and meta-analysis (25) reported that the prevalence of DED symptoms in visual display terminal (VDT) users is 62.6–85.8%, much higher than in the general population. The ADEC cohort’s high proportion of digital natives provides an opportunity to investigate the long-term cumulative effects of chronic screen exposure on DED pathogenesis—an area of great clinical relevance given the global increase in digital device use, especially after the COVID-19 pandemic (which led to a sharp rise in remote work and online learning) (25). Future longitudinal follow-up of the ADEC cohort will be able to evaluate the dose–response relationship between digital device use duration/frequency and DED progression, and evaluate the potential role of behavioral interventions (e.g., blink training, screen distance adjustment) in DED prevention and symptom management in digital natives.

### Key subpopulations and multi-omics: the innovation of ADEC

A major strength of the ADEC study is the systematic inclusion of high-risk DED subpopulations (digital natives, autoimmune disease patients, perimenopausal/postmenopausal women) and the standardized collection and storage of tear fluid samples for multi-omics analysis (lipidomics, metagenomics, proteomics). Autoimmune disease patients (5.13% in the cohort) are a key high-risk group for DED, as autoimmune diseases (e.g., Sjögren’s syndrome, rheumatoid arthritis) induce lacrimal and salivary gland dysfunction via Th17/Treg imbalance and IL-17-mediated epithelial damage (10, 11). The ADEC cohort’s collection of tear fluid samples from these patients will enable future multi-omics analysis to identify specific molecular biomarkers (e.g., inflammatory cytokines, lipid metabolites) of autoimmune-related DED, providing a basis for future biomarker discovery and targeted research.

In addition, the ADEC study incorporated standardized symptom and sign assessments for DED diagnosis, consistent with contemporary diagnostic methodology (34). For analytic purposes, DED was summarized as a participant-level primary outcome, and SDE, AD phenotype, and MGD phenotype were assessed using symptom-plus-sign combinations, which enables phenotype-specific evaluation of DED characteristics and future treatment research. For example, AD is mainly related to tear secretion deficiency (e.g., lacrimal gland hypofunction), while MGD is related to increased tear evaporation (e.g., meibomian gland dysfunction), and the two subtypes require different treatment strategies (e.g., artificial tears for AD, warm compresses and lipid-based artificial tears for MGD) (35). The ADEC’s subtype-specific prevalence data provide baseline evidence for future phenotype-targeted DED research in China.

### Strength and limitations of the study

The ADEC study demonstrates excellent data reliability as evidenced by several key metrics. The high response rate of 91.5% indicates good community engagement and reduces nonresponse concerns. The longitudinal follow-up framework supports future analyses of DED incidence and progression, although the present report is restricted to baseline data. Furthermore, the high inter-rater reliability (ICC or weighted Kappa >0.8) ensures the consistency and accuracy of clinical assessments across different examiners.

The study also has limitations. First, the study population was recruited from urban Dongcheng District, Beijing, with a high proportion of older adults and women. Although age–sex standardization was performed, generalization to other regions of China or rural populations should be made cautiously. Second, some data relied on self-reporting, including electronic device use duration, smoking, and alcohol consumption, which may be subject to recall bias and social desirability bias. Third, the analyses presented here are enrollment-stage baseline analyses; therefore, observed associations should not be interpreted as causal relationships or drivers of disease progression.

## Conclusion

In this population-based cohort study conducted in urban Beijing, DED was common among community-dwelling adults, with substantial variation across age, sex, and dry eye-related phenotypes. The baseline ADEC data support the multifactorial nature of DED and establish a platform for future longitudinal analyses of environmental, behavioral, systemic, psychological, and molecular contributors to dry eye disease.

## Data Availability

The raw data supporting the conclusions of this article will be made available by the authors, without undue reservation.
